# Clinical characteristics of aseptic meningitis induced by intravenous immunoglobulin in patients with Kawasaki disease

**DOI:** 10.1186/1546-0096-9-28

**Published:** 2011-09-14

**Authors:** Yasushi Kemmotsu, Tomotaka Nakayama, Hiroyuki Matsuura, Tsutomu Saji

**Affiliations:** 1 Department of Pediatrics, School of Medicine, Faculty of Medicine, Toho University, Tokyo 143-8541, Japan

**Keywords:** Kawasaki disease, intravenous immunoglobulin, aseptic meningitis

## Abstract

**Background:**

Aseptic meningitis is a serious adverse reaction to intravenous immunoglobulin (IVIG) therapy. We studied the clinical characteristics of patients with acute Kawasaki disease (KD) who developed IVIG-induced aseptic meningitis.

**Methods:**

A retrospective analysis of the medical records of patients with KD who developed aseptic meningitis after IVIG treatment was performed.

**Results:**

During the 10-year period from 2000 through 2009, among a total of 384 patients with Kawasaki disease, 4 (3 females and 1 male; age range, 19-120 months) developed aseptic meningitis after IVIG. All 4 developed aseptic meningitis within 48 hours (range, 25-40 hours) of initiation of IVIG. The analyses of cerebrospinal fluid (CSF) revealed elevated white blood cell counts (22-1,248/μL) in all 4 patients; a predominance of polynuclear cells (65%-89%) was noted in 3. The CSF protein level was elevated in only 1 patient (59 mg/dL), and the glucose levels were normal in all 4 patients. Two patients were treated with intravenous methylprednisolone; the other 2 children were observed carefully without any special therapy. All patients recovered without neurological complications.

**Conclusions:**

In our patients with Kawasaki disease, aseptic meningitis induced by IVIG occurred within 48 hours after initiation of IVIG, resolved within a few days, and resulted in no neurological complications, even in patients who did not receive medical treatment.

## Background

Intravenous immunoglobulin (IVIG) is a blood product that is widely used in the treatment of a number of medical conditions, including immunodeficiency disorders, inflammatory diseases, and autoimmune diseases.

Kawasaki disease (KD) is a self-limited systemic vasculitis syndrome of childhood that was first reported by Tomisaku Kawasaki in 1967 [1]. Patients typically develop a fever, bulbar conjunctival injection, changes in the oropharyngeal mucosa and peripheral extremities, cervical lymphadenopathy, and a polymorphous rash. Coronary aneurysm and myocardial infarction are the most serious complications of this disease. In Japan, there are approximately 10,000 incident cases per year [2]. The etiology of the disease is not well understood, but high-dose IVIG is known to prevent the coronary complications [3,4].

There have been a number of reports regarding IVIG-induced adverse reactions, including mild reactions such as tachycardia, headache, facial flushing, nausea, diarrhea, and rash, as well as serious adverse reactions such as anaphylaxis, acute renal failure, and thromboembolic events [5]. Aseptic meningitis is a neurologic adverse event that can be caused by IVIG. Although there have been case reports describing IVIG-induced aseptic meningitis, few studies have described the characteristics of a group of such patients. In this study, we describe the clinical and laboratory characteristics of IVIG-induced aseptic meningitis in 4 patients with KD.

## Patients and methods

### Patients

To investigate the clinical characteristics of IVIG-induced meningitis in KD patients, we retrospectively reviewed the medical records of patients who were admitted to our university hospital during the 10-year period from 2000 through 2009. All patients met the Japanese criteria for typical KD on admission. They were treated with oral aspirin and 1 or 2 g/kg of IVIG, the latter of which was administered over 12 or 24 hours, respectively. The IVIG products were freeze-dried sulfonated (Kenketsu Venilon^®^-I, Chemo-Sero-Therapeutic Research Institute, Kumamoto, Japan) and freeze-dried, polyethylene glycol (PEG) -treated (Kenketsu Glovenin^®^-I, Nihon Pharmaceutical Co, Ltd, Tokyo, Japan) human normal immunoglobulin. Testing of the CSF was done soon after the diagnosis of suspected IVIG-induced meningitis, and a diagnosis of meningitis was made on the basis of clinical symptoms such as fever and headache, meningeal irritation signs, and CSF pleocytosis. A final diagnosis of aseptic meningitis was made by negative bacterial culture results.

## Results

### Characteristics of the study population and IVIG products

A total of 384 patients with KD were admitted to our hospital; 4 developed aseptic meningitis after IVIG. Table 1 shows the background characteristics of these 4 patients. Three were females older than 5 years. The other patient was a 1-year-old male. Their serum C-reactive protein (CRP) levels and white blood cell counts before IVIG treatment were 3.3-5.5 mg/dL and 6,500-27,100/μL, respectively. Sulfonated immunoglobulin was given to 2 patients, and a polyethylene glycol-treated product was given to the other 2 patients. Two patients were treated with 1 g/kg IVIG, and the other 2 received 2 g/kg IVIG. There were no adverse reactions during the IVIG administration in any of the patients.

**Table 1:** Background characteristics of the patients

Age	Sex	KD criteria	CRP(mg/dL)/WBC(/μL) on admission	IVIG product and dose	Day on IVIG
1 y	male	5/6	5.5/6,500	PEG-treated 2 g/kg	8
6 y	female	5/6	7.1/15,600	Sulfonated 1 g/kg	5
7 y	female	6/6	7.8/27,100	Sulfonated 2 g/kg	5
10 y	female	6/6	3.3/9,900	PEG-treated 1 g/kg	4

KD = Kawasaki disease; CRP = C-reactive protein; WBC = white blood cell; IVIG = intravenous immunoglobulin; PEG = polyethylene glycol.

### Clinical course and laboratory findings

All 4 patients responded well to initial IVIG: their fevers ceased and the clinical symptoms of KD improved. Table 2 shows the clinical course of the patients. Aseptic meningitis developed within 48 hours (range, 25-40 hours) after initiation of IVIG. All 4 patients developed a sudden, severe fever. Their recorded highest body temperatures were 38.0, 38.7, 38.8, and 39.1°C. The 3 females complained of headache, and the 1-year-old male was irritable and vomited frequently. On physical examination, there were typical signs of meningeal irritation, including neck rigidity, Kernig's sign, and Brudzinski's sign. Table 3 shows the CSF findings of the 4 patients. The initial pressure was recorded in 1 patient and was mildly elevated (24 cm H_2_O). The analyses of the CSF revealed elevated white blood cell counts (22-1,248/μL) in all 4 patients, 3 of whom were neutrophil-predominant (65%-89%). The CSF protein level was elevated in only 1 patient (59 mg/dL), and the glucose levels were normal in all 4 patients (51-77 mg/dL). The CSF chloride and lactate dehydrogenase (LDH) levels were measured in 3 patients and were normal (123-128 mEq/L and 33-40 U/L, respectively). In addition, the results of CSF bacterial culture were negative in all patients. There was no worsening of inflammatory markers, ie, serum CRP and peripheral white blood cell counts, at the onset of meningitis (mean ± SD CRP: 4.3 ± 4.1 mg/dL, WBC: 9,300 ± 7,700/μL), as compared with the levels at admission (mean ± SD CRP: 5.9 ± 2.0 mg/dL, WBC: 14,800 ± 9,000/μL). Two patients were treated with a single dose of 15 mg/kg of intravenous methylprednisolone; the other 2 patients recovered without medical treatment. Fever and signs of meningeal irritation disappeared in 1 or 2 days, and no patient developed any neurological complications such as seizures or disturbances in consciousness. There was no recurrence of KD in any of the patients, and all four patients were discharged without coronary artery aneurysms.

**Table 2:** The clinical course of the patients.

Patient	Time from start of IVIG to onset, hrs	Treatment	Time to recovery
1 y male	33	15 mg/kg mPSL	1 day
6 y female	40	15 mg/kg mPSL	2 days
7 y female	25	None	2 days
10 y female	31	None	1 day

IVIG = intravenous immunoglobulin; mPSL = methylprednisolone.

**Table 3:** Cerebrospinal fluid findings

Patient	Cells (/μL)	Glucose (mg/dL)	Protein (mg/dL)	LDH (U/L)
1 y male	1,248 (P 89%)	51	59	39
6 y female	120 (P 13%)	54	23	33
7 y female	648 (P 83%)	77	30	40
10 y female	21 (P 65%)	52	37	NT

LDH = lactate dehydrogenase; P = polynuclear cells; NT = Not tested.

## Discussion

Aseptic meningitis after IVIG was first reported in 1988 [6]. Since then, there have been similar case reports of IVIG-induced meningitis in patients with medical conditions such as idiopathic thrombocytopenic purpura (ITP), myasthenia gravis, and inflammatory demyelinating neuropathy [7-9]. There has previously been only 1 case report describing this complication in a patient with KD [10].

The rate of aseptic meningitis after IVIG was 1% (4 of 384) in this study, but the frequency varies widely, from 0% to 11%, in reports of patients with different underlying diseases [11,12]. It was also reported that the development of aseptic meningitis was not correlated with the patient age or the type of underlying neuromuscular disease [12].

Hamrock reported that most patients who developed aseptic meningitis received 2 g/kg of IVIG, and that meningitis did not occur in any of their patients receiving a standard replacement dose of IVIG for a congenital immunodeficiency [5]. All of our patients received high-dose IVIG at a dose of 1 or 2 g/kg. Our patients almost equally received sulfonated IVIG or PEG-treated IVIG, and 2 patients in each group (total 4) developed meningitis, thus indicating that there are no apparent differences in the effects of sulfaonated or PEG-treated IVIG with regard to the development of meningitis. In this study, patients were exposed to either sulfonated IVIG or PEG-treated IVIG, but not to products manufactured by other processes such as cold ethanol Cohn fractionation/ultrafiltration, ion exchange, or low-PH treatment. The inability to further explore the possible etiological factors related to specific IVIG brand or manufacturing lots may be a limitation of this study. There were no obvious differences of clinical and laboratory data, including the severity of KD on admission, day of initiating IVIG, or changes of inflammatory markers after IVIG between patients who developed meningitis and those who did not.

In the present study, aseptic meningitis developed within 25 to 40 hours after initiation of IVIG. In previous case reports, most patients also developed meningitis within 48 hours of beginning IVIG. Although all of our patients developed a fever and typical meningeal irritation signs, it may be possible that milder cases of aseptic meningitis could be misdiagnosed as IVIG- refractory KD, since the onset of fever after completion of IVIG therapy is often interpreted as recrudescence of KD. It is important to consider the possibility of IVIG-induced meningitis with careful physical examinations to avoid unnecessary therapies, such as additional IVIG, steroids, and infliximab.

CSF examinations revealed neutrophilic pleocytosis in 3 of our 4 patients, slight elevation of the protein level in 1 patient, and normal glucose levels in all 4 patients. These findings were similar to those of previous reports. The analysis of the CSF in patients with aseptic meningitis usually shows pleocytosis with neutrophil predominance, normal or slightly elevated protein, and normal glucose levels. It may therefore be difficult to differentiate IVIG-induced meningitis from viral meningitis by the CSF findings, as it has been reported that the CSF protein levels are normal to mildly elevated, glucose levels are normal to slightly depressed, and neutrophil predominance is also seen in pediatric patients with viral meningitis [13,14].

All of our patients recovered without developing any neurological complications. Two were treated with intravenous methylprednisolone, and the other 2 were monitored without medical treatment. Jayabose et al. reported that children with ITP who were given prednisone had a lower risk of neurological complications after IVIG [15]. However, it has also been reported that such symptoms are self-limiting, and that there is no specific therapy that shortens the duration of symptoms. Thus, it may be advisable to carefully observe such patients and avoid systemic therapy [5]. In our study, there were no obvious differences in the clinical courses between patients treated with intravenous methylprednisolone and those who received no medical treatment, which suggests that systemic steroid administration is not beneficial for IVIG-induced meningitis.

The mechanisms underlying IVIG-induced meningitis are not clear. One possible cause is an allergic hypersensitivity reaction caused by direct entry of the IVIG preparation into the CSF compartment. This is supported by the fact that CSF eosinophilia has been observed in some patients [11]. In our study, one patient exhibited peripheral eosinophilia (11% of the total 5,800/μL white blood cells) but CSF eosinophilia was not observed in any of our patients. None of our patients developed exanthema after IVIG. Although our patients received no pre-treatment, it may be useful to give antihistamines prior to IVIG if allergic reaction is one of the mechanisms responsible for IVIG-induced meningitis. Recently, it was reported that there were increased levels of CSF monocyte chemoattractant protein-1 (MCP-1) in ITP patients with IVIG-induced meningitis, which suggests a role for monocytes in the inflammation of the meninges [16]. On the other hand, Jarius et al. reported that aseptic meningitis was frequently associated with neutrophillic pleocytosis in the CSF and *in vivo *activation of TNF-α-primed neutrophils by atypical antineutrophil cytoplasmic antibodies in IVIG might contribute to aseptic meningitis [17]. In our present study, the CSF cytokines or chemokines were not measured.

Meningitis is also a known complication of KD. Dengler et al reported that one-third of patients with KD who underwent a lumbar puncture had CSF pleocytosis with mononuclear cell predominance [18], which is in contrast to the polynuclear cell predominance observed in IVIG-induced meningitis. Meningitis as a complication of KD usually occurs early in the course of the disease and improves after KD treatment, which is mainly IVIG therapy [19]. Table 4 shows a comparison between IVIG- and KD-induced meningitis. It is not difficult to differentiate IVIG-induced meningitis from aseptic meningitis complicating KD, as both the time of onset and CSF findings differ.

**Table 4:** A comparison between IVIG- and KD-induced meningitis

	Meningitis due to IVIG	Meningitis due to KD
Appearance	Within 48 hrs after IVIG	Early in the stage, before IVIG
Clinical findings	Typical meningeal signs	Can lack meningeal signs
CSF findings	Polynuclear cell predominance	Mononuclear cell predominance
Effective therapy	No special therapy	Therapy for KD

IVIG = intravenous immunoglobulin; KD = Kawasaki disease; CSF = Cerebrospinal fluid.

## Conclusions

In conclusion, IVIG-induced meningitis developed within 48 hours of initiating IVIG and resolved in a few days, without neurological complications, and systemic steroid administration was not beneficial in our patients. Further investigations of the pathophysiology of IVIG-induced meningitis, including a detailed analysis of the underlying mechanisms, are needed.

## Competing interests

The authors declare that they have no competing interests.

## Authors' contributions

YK contributed by taking care of the patients. All authors contributed to the analysis and interpretation of the data. All authors read and approved the final manuscript.
